# American Public Health Association

**Published:** 1886-01

**Authors:** 


### Thirteenth Annual Meeting of the American Public Health Association.

The meeting of this association was held at Washington, December 8–12, 1885, more than thirty States being represented. Congress was petitioned for such legislation as would secure to the whole country some central body, with power and money sufficient to investigate the sources of diseases, especially those known to be preventable. Numerous valuable papers were read; a first prize was awarded to Dr. Sternberg, U.S.A., for an essay on disinfection and individual prophylaxis, and a second prize to Dr. Lincoln for an essay on school hygiene, no first prize being awarded. Dr. H. P. Walcott was elected President of the association for the coming year.

